# The impact of chitosan on the early metabolomic response of wheat to infection by *Fusarium graminearum*

**DOI:** 10.1186/s12870-022-03451-w

**Published:** 2022-02-19

**Authors:** Myriam Deshaies, Nadia Lamari, Carl K. Y. Ng, Patrick Ward, Fiona M. Doohan

**Affiliations:** 1 UCD School of Biology and Environmental Science, UCD Centre for Plant Science, and UCD Earth Institute, University College Dublin, O’Brien Centre for Science, Belfield, Dublin, Ireland; 2 Envirotech Innovative Products Ltd, NovaUCD, Belfield Innovation Park, Belfield, Dublin, Ireland; 3 Philip Morris International, Quai Jeanrenaud 3, 2000 Neuchatel, Switzerland

**Keywords:** *Fusarium graminearum*, Wheat, Chitosan, Antifungal activity, Disease severity, Untargeted metabolomics, UHPLC-QTOF-MS

## Abstract

**Background:**

Chitosan has shown potential for the control of Fusarium head blight (FHB) disease caused by *Fusarium graminearum.* The objective of this study was to compare the effect of chitosan hydrochloride applied pre- or post-fungal inoculation on FHB and to better understand its’ mode of action via an untargeted metabolomics study.

**Results:**

Chitosan inhibited fungal growth *in vitro* and, when sprayed on the susceptible wheat cultivar Remus 24 hours pre-inoculation with *F. graminearum,* it significantly reduced the number of infected spikelets at 7, 14 and 21 days post-inoculation. Chitosan pre-treatment also increased the average grain weight per head, the number of grains per head and the 1000-grain weight compared to the controls sprayed with water. No significant impact of chitosan on grain yield was observed when the plants were sprayed 24 hours post-inoculation with *F. graminearum,* even if it did result in a reduced number of infected spikelets at every time point. An untargeted metabolomic study using UHPLC-QTOF-MS on wheat spikes revealed that spraying the spikes with both chitosan and *F. graminearum* activated known FHB resistance pathways (e.g. jasmonic acid). Additionally, more metabolites were up- or down-regulated when both chitosan and *F. graminearum* spores were sprayed on the spikes (117), as compared with chitosan (51) or *F. graminearum* on their own (32). This included a terpene, a terpenoid and a liminoid previously associated with FHB resistance.

**Conclusions:**

In this study we showed that chitosan hydrochloride inhibited the spore germination and hyphal development of *F. graminearum in vitro*, triggered wheat resistance against infection by *F. graminearum* when used as a pre-inoculant, and highlighted metabolites and pathways commonly and differentially affected by chitosan, the pathogen and both agents. This study provides insights into how chitosan might provide protection or stimulate wheat resistance to infection by *F. graminearum*. It also unveiled new putatively identified metabolites that had not been listed in previous FHB or chitosan-related metabolomic studies.

**Supplementary Information:**

The online version contains supplementary material available at 10.1186/s12870-022-03451-w.

## Background

Wheat production is threatened by fungal diseases that are estimated to detrimentally affect grain yields by 15-20% per annum [1, 2]. Fusarium head blight (FHB), also called Fusarium ear blight or scab, is one of the major fungal diseases affecting global wheat production. The disease also affects the yield of oat, barley and maize. In wheat, FHB is most commonly caused by the fungi *Fusarium graminearum* and *F. culmorum,* but also by other *Fusarium* species such as *F. avenaceum* and *F. poae* [3]. *F. graminearum* and *F. culmorum* both cause significant yield losses and contaminate grain with harmful mycotoxins, including trichothecenes and zearalenone [3]. *Fusarium* species can infect the seeds and seedlings of new plants growing in the field, causing Fusarium seedling blight (FSB) [4, 5] and Fusarium root rot (FRR) diseases [6].

Several management strategies, such as using seed and foliar fungicides, sowing less susceptible cultivars, and practicing tillage and crop rotation, usually need to be combined to retard the development of FHB disease [7, 8]. It is recommended to use less susceptible cultivars and to spray the plants with fungicides during the early flowering period before infection and repeatedly after flowering. However, genetic resistance can only provide moderate disease control and the effectiveness of fungicides highly depends on the timing of application and on weather conditions. Additionally, the use of fungicides is often controversial because of the potential hazards for human health and the environment, and there is a heightened risk of pathogens developing resistance to these fungicides. Biostimulants are now being widely developed to replace or reduce the use of synthetic fungicides. One such biostimulant is chitosan; it is a polysaccharide derived from the deacetylation of chitin, which is often extracted from crustacean shells and also exists in fungal cell walls. Chitosan was previously shown to reduce the severity of FHB [9–12], FSB [4, 5] and FRR [6] diseases in wheat and barley. It inhibited *F. graminearum* growth *in vitro* and under greenhouse conditions when it was sprayed onto pathogen-inoculated spikes [10, 11]. It decreased the severity of FHB and the DON content in grains when applied as a head spray treatment 24 hours pre-pathogen inoculation [9] and in spikes when applied 48 hours pre-pathogen inoculation [12]. Chitosan used as a seed or soil amendment treatment also reduced the seedling blight disease severity on wheat and barley inoculated with *F. culmorum* [5]. Therefore, chitosan’s capacity to reduce the impact of FHB and FSB is well established. In the study comparing the efficacy of chitosan against FHB when applied just before fungal inoculation vs 3 or 5 days post-inoculation, chitosan was found to be more effective at reducing the disease severity when applied as a pre-inoculation treatment rather than when applied 3 or 5 days post-inoculation [10]. However, no study had previously compared the pre- vs post-inoculation effects of chitosan on development of both grain and disease symptoms.

Metabolomic studies have elucidated impact of chitosan [13] and the fungus [14–16] on wheat, but we have limited knowledge as to how the combined effect of these agents influences host defences. Chitosan is known for its ability to (i) directly inhibit fungal growth by damaging fungal cell walls and membranes, (ii) strengthen plant tissues by stimulating lignin deposition, to elicit plant production of antimicrobial phenolic compounds and (iii) create a physical barrier between the plant tissue and the pathogens [17]. Only a few studies have focused on elucidating the biochemical effects of chitosan on wheat and against *Fusarium* infection. It was shown that chitosan applied to wheat seeds stimulated the production of phenols involved in lignification and having antimicrobial activity [6]. The stimulation of the synthesis of phenols having antimicrobial activity, especially ferulic acid, was also reported in another study as well as an increase in lignin content in the leaves after seed treatment with chitosan [4]. Chitosan hydrochloride applied on the flag leaves of wheat was also shown to decrease mycotoxin accumulation and to activate genes involved in systemic acquired resistance [12].

The overall aim of this study was to improve our understanding of the biochemical mechanisms involved in chitosan-mediated control of FHB disease. The first objective was to verify that chitosan hydrochloride had antifungal activity against *F. graminearum in vitro* and to compare its’ efficacy as a spray treatment to reduce the deleterious effects of FHB, both visually and on development of grain, when applied pre- vs post-fungal inoculation. Thereafter, untargeted UHPLC-QTOF-MS analysis was used to investigate the impact of chitosan on the secondary metabolite profiles of wheat when it was applied alone or as a pre-fungal inoculation treatment and in comparison with the impact of the fungal infection alone. Based on this study, we discuss the potential mechanisms through which chitosan reduces the severity of FHB disease.

## Results

### Chitosan inhibits the growth of *F. graminearum in vitro*

**Fig. 1:**
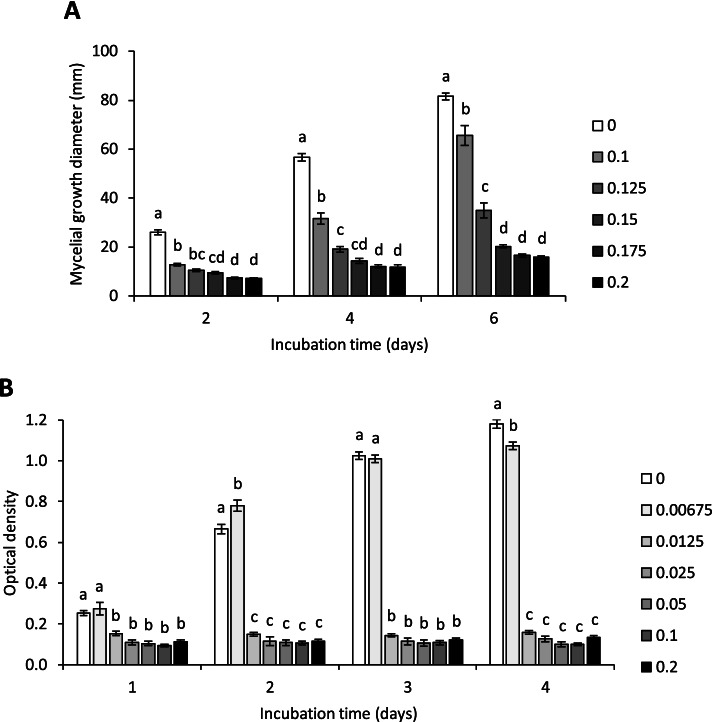
*In vitro* dose response effects of chitosan on the growth of *F. graminearum* GZ3639. **A** Potato Dextrose Agar (PDA) was prepared with chitosan to final concentrations of 0.0, 0.1, 0.125, 0.15, 0.175 or 0.2% (w v^-1^) and was inoculated with a plug of *F. graminearum*. The mycelial growth diameter (mm) was measured every 2 days for 6 days of incubation. **B** Potato Dextrose Broth (PDB) was prepared with chitosan to final concentrations of 0.0, 0.00675, 0.0125, 0.025, 0.05, 0.1 or 0.2% (w v^-1^) and was inoculated with conidia of *F. graminearum*. The (**B**) optical density was measured every day for 4 days after incubation. Error bars represent the standard error of the means. Different letters above the data sets indicate that the data are statistically significantly different according to one-way ANOVA tests performed for each incubation time (*P* ≤ 0.05)

Both solid and liquid culture antifungal activity tests were performed to evaluate the response of *F. graminearum* to a range of concentrations of chitosan incorporated into Potato Dextrose Agar (PDA) or Potato Dextrose Broth (PDB). The results of the two experiments revealed that the fungal growth was inhibited with increasing concentrations of chitosan in the growth media (Fig. 1A and B and Fig. S1A and B). Even the lowest concentration of chitosan tested in solid and liquid cultures significantly inhibited growth. By 6 days post-inoculation (dpi), solid PDA amendment with 0.1% chitosan resulted in a 20% reduction compared with the control (*P* = 0.040), increasing to 85% inhibition with 0.2% chitosan (*P* ≤ 0.001; Fig. S1A). By 4 dpi, liquid PDB amendment with 0.00675% chitosan resulted in 9% growth inhibition compared to the control (*P* = 0.015) and reached 91% inhibition with 0.2% chitosan (*P* ≤ 0.001; Fig. S1B).

### Chitosan reduces the severity of FHB when applied pre- or post-fungal inoculation

**Fig. 2:**
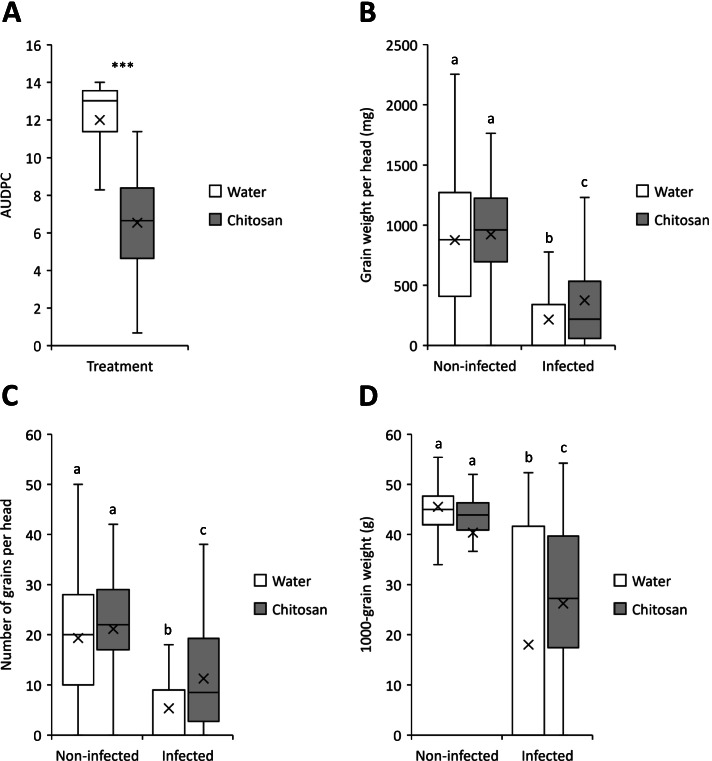
Box plot distribution of the effect of spraying wheat heads with chitosan pre-inoculation on the development of Fusarium head blight disease and grain yield. The heads of wheat cv. Remus were sprayed at mid-anthesis with water (control) or 0.2% chitosan (w v^-1^) and were spray-inoculated 24 hours before with 0.02% Tween 20 (mock) or 10^5^ spores ml^-1^ *F. graminearum* GZ3639 in 0.02% Tween 20. The (**A**) Area Under Disease Progress Curve (AUDPC) was calculated based on the percentage of infected spikelets measured at 7, 14 and 21 dpi from five replica trials. The (**B**) average grain weight per head (mg), **C** number of grains per head and (**D**) 1000-grain weight (g) were measured from three replica trials. Medians are indicated by solid lines; × represents mean. **A** Asterisks above the data sets indicate that the data are statistically significantly different from the mock water treatment according to Kruskal-Wallis tests (*** = *P* ≤ 0.001). **B**, **C** and **D** Different letters above the data sets indicate that the data are statistically significantly different according to Kruskal-Wallis tests (*P* ≤ 0.05)

**Fig. 3:**
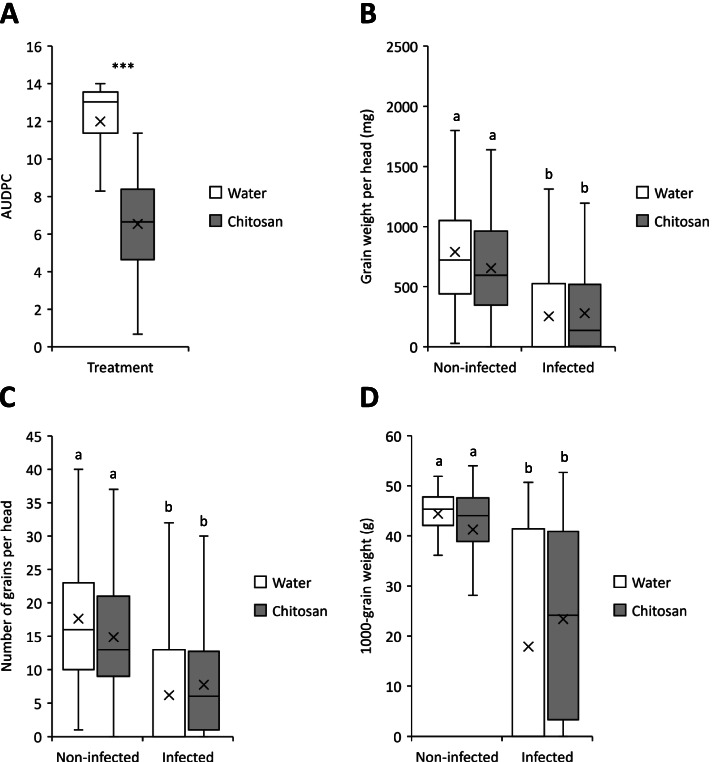
Box plot distribution of the effect of spraying wheat heads with chitosan post-inoculation on the development of Fusarium head blight disease and grain yield. The heads of wheat cv. Remus were spray-inoculated at mid-anthesis with 0.02% Tween 20 (mock) or 10^5^ spores ml^-1^ *F. graminearum* GZ3639 in 0.02% Tween 20 and were sprayed 24 hours later with water (control) or 0.2% chitosan (w v^-1^) and the (**A**) Area Under Disease Progress Curve (AUDPC) was calculated based on the percentage of infected spikelets measured at 7, 14 and 21 dpi from five replica trials. The (**B**) average grain weight per head (mg), **C** number of grains per head and (**D**) 1000-grain weight (g) were measured from three replica trials. Medians are indicated by solid lines; × represents mean. A Asterisks above the data sets indicate that the data are statistically significantly different from the mock water treatment according to Kruskal-Wallis tests (*** = *P* ≤ 0.001). **B**, **C** and **D** Different letters above the data sets indicate that the data are statistically significantly different according to Kruskal-Wallis tests (*P* ≤ 0.05)

After verifying that chitosan could inhibit the growth of *F. graminearum in vitro*, the next objective of the research was to validate that it could be used to control FHB disease caused by *F. graminearum* in wheat, either as a preventative (applied before infection) or curative treatment (applied after infection). The wheat heads were sprayed with chitosan 24 h before fungal inoculation to evaluate its’ effectiveness as a preventative treatment, or they were sprayed with chitosan 24 h after fungal inoculation to assess its’ curative effects. Whether it was applied 24 h pre- (Fig. S2A) or post-fungal inoculation (Fig. S2B), chitosan significantly reduced the percentage of infected spikelets observed at 7, 14 and 21 dpi compared to mock water treatment (*P* ≤ 0.001 at all time points). Chitosan also reduced disease build up over time, assessed as the Area Under Disease Progress Curve (AUDPC): compared with water it reduced AUDPC from 13.0 to 6.7 (median values) when applied pre-fungal inoculation (*P* ≤ 0.001; Fig. 2A), and from 12.5 to 6.3 (median values) when applied post-fungal inoculation (*P* ≤ 0.001; Fig. 3A).

In the absence of chitosan treatment, *Fusarium* inoculation significantly reduced the average grain weight per head (*P* ≤ 0.001; Fig. 2B and 3B), the number of grains per head (Fig. 2C and 3C) and the 1000-grain weight (*P* ≤ 0.001; Fig. 2D and 3D) (for mock-treated heads sprayed with water pre- or post-fungal inoculation). In the absence of the pathogen, chitosan did not impact the grain weight per head, the number of grains per head, nor the 1000-grain weight (compared with water-treated controls), whether it was applied pre-mock inoculation (*P* = 0.774, 0.385 and 0.135, respectively) or post-mock inoculation (*P* = 0.150, 0.133 and 0.123, respectively).

Applied pre-fungal inoculation, chitosan reduced the loss of grain weight caused by the pathogen. It significantly increased the average grain weight per head by 75% (*P* = 0.001; Fig. 2B), the number of grains per head by 112% (*P* ≤ 0.001; Fig. 2C) and the 1000-grain weight by 46% (*P* = 0.032; Fig. 2D), as compared to water treatment. However, it had no significant effect on the grain weight (*P* = 0.387; Fig. 3B), the number of grains (*P* = 0.065; Fig. 3C) and the 1000-grain weight (*P* = 0.118; Fig. 3D), when applied post-inoculation.

Taken together, the FHB trial results showed that chitosan decreased the FHB disease severity compared with water when it was applied pre- or post-inoculation and reduced the yield loss caused by the disease when it was applied 24 h pre-fungal inoculation. Hence, the results corroborated previous findings wherein non-water-soluble chitosan or chitosan hydrochloride were used as a preventative treatment against FHB caused by *F. culmorum* in wheat and barley [9] or as a curative treatment against FHB caused by *F. graminearum* in wheat [10–12]. Beyond that, they also showed that its’ ability to limit the negative effects of *Fusarium* on grain development were restricted to pre- versus post-pathogen application.

### Metabolite profiling and annotation

**Fig. 4:**
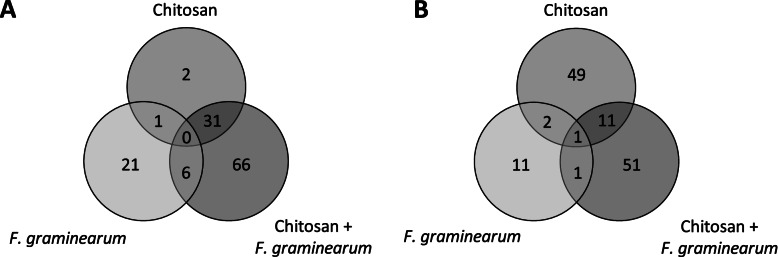
Representation of the number of features of interest (potential metabolites). The features of interests were delineated by Volcano plot analysis and correspond to potential metabolites differentially regulated according to the fold change (FC, cut-off value 1.5) and results of the t-tests (*P* ≤ 0.05) for each category comparison. The samples corresponding to the 6- and 24-hour time points were pooled together for the statistical analysis. Three comparisons were performed: chitosan (samples W_T compared with C_T, number of features significantly up- or down-regulated by chitosan), *F. graminearum* (samples W_T compared with W_F, number of features significantly up- or down-regulated by *F. graminearum*) and chitosan + *F. graminearum* (samples W_T compared with C_F, number of features significantly up- or down-regulated by chitosan + *F. graminearum*). The Venn diagrams represent (**A**) the number of features up-regulated and (**B**) the number of features down-regulated compared with W_T

Volcano plots were produced which delineated the potential features (metabolites) differentially regulated in wheat spikes (Fold change, FC, of at least 1.5; *P* ≤ 0.05) in response either to chitosan (comparing W_T vs C_T), *F. graminearum* (W_T vs W_F) or the combination of chitosan and *F. graminearum* (W_T vs C_F) (Fig. S3A, B and C, respectively). The details of the numbers of up and down-regulated features of interest are represented with Venn diagrams comprising the different categories (Fig. 4). A total of 235 features of interest were delineated but not all could be annotated (Tables 1 and 2). For each comparison, the annotation of at least 20 features (10 with the highest up-regulation and 10 with the highest down-regulation, where possible) was applied using full scan fragmentation data. In total, 92 features were tested to validate their *m/z* value before trying to annotate them. Their theoretical *m/z* values (obtained by processing the data via Galaxy Workflow4Metabolomics and MetaboAnalyst) were compared with the experimental *m/z* values (obtained by processing the chromatograms with the molecular feature extraction algorithm via MassHunter Workstation software – Qualitative analysis).

**Table 1:** List of features differentially produced in response to either chitosan (down-regulated only), *F. graminearum* or both agents together^a^

Feature	RT (min)	Measured ***m/z*** (-)	Neutral ***m/z***	FC	Putative identification	Exact ***m/z***	Error (ppm)
**Downregulated by chitosan**							
M395T189	3.30	395.0786	396.0859	0.42	Apigenin triacetate* 3,5,6-Trimethoxy-3',4'-methylene-dioxyfurano[2,3:7,8]flavone	396.0845 -	3 -
**Upregulated by** ***F. graminearum***							
M293T380	6.57	293.0984	294.1057	2.27	Distichonic acid	294.1063	2
M365T570	9.60	365.1800	366.1873	1.72	7-Methyl-3-methylene-1,2,6,7-octanetetrol 2-glucoside p-Menthane-1,2,8,9-tetrol 9-glucoside	366.1890 -	4 -
M327T1531	25.93	327.217	328.2243	2.04	9,12,13-Trihydroxyoctadecadienoic acid (TriHODE) 9-hydroperoxy-12,13-epoxy-10-octadecenoic acid 11-hydroperoxy-12,13-epoxy-9-octadecenoic acid	328.2250 - -	2 - -
**Downregulated by** ***F. graminearum***							
M310T230	3.84	310.0993	311.1066	0.63	Cinnamoyl beta-D-glucoside	310.1012	4
M403T278	4.64	403.0935	404.1008	0.61	Mollicellin D	404.1027	4
**Upregulated by the combined treatment with chitosan +** ***F. graminearum***							
M497T463	7.74	497.2050	498.2123	3.04	Iridodial glucoside tetraacetate 8-Epiiridodial glucoside	498.2101 -	4 -
M663T1079	18.02	663.3716	664.3789	2.54	Phytolaccoside B Medicagenic acid 3-O-beta-D-glucoside Elatoside G	664.3823 - -	5 - -
M595T1405	23.42	595.2960	596.3033	2.57	Salannin	596.2985	7
M565T1500	25.01	565.3217	566.3290	3.38	25-Cinnamoyl-vulgaroside	566.3244	8
M565T1532	25.50	565.3215	566.3288	2.89	25-Cinnamoyl-vulgaroside	566.3244	7
**Downregulated by the combined treatment with chitosan +** ***F. graminearum***							
M434T773	12.82	434.2088	435.2161	0.40	Cadabicine	435.2158	0.7
M469T1211	20.46	469.1836	470.1909	0.48	Limonin Zapoterin Butyrylmallotochromene Drummondin A	470.1941 - - -	6 - - -

The exact *m/z* value is the monoisotopic mass of the compound suggested for annotation. The error is the relative difference between the experimental neutral *m/z* value and the exact *m/z*. The asterisk indicates the metabolite that most likely corresponds to the feature

*RT* retention time, *FC* fold change

^a^ Features were annotated based on METLIN and literature review

**Table 2:** List of features differentially produced in response to more than one treatment combination^a^

Feature	RT (min)	Measured ***m/z*** (-)	Neutral ***m/z***	FC	Putative identification	Exact ***m/z***	Error (ppm)
**Upregulated by both (i) chitosan and (ii) chitosan +** ***F. graminearum***							
M218T324	5.32	218.0830	219.0903	3.25 3.17	Nigellimine N-oxide Alpha-hydroxy-1-methyl-1H-indole-3-propanoic acid Alpha-methoxy-1H-indole-3-propanoic acid	219.0895 - -	3 - -
M533T416	6.93	533.2163	534.2236	2.40 3.94	Sarothralin	534.2254	3
M687T1403	23.39	687.3585	688.3658	2.99 3.12	Callichiline	688.3625	4
**Upregulated by both (i)** ***F. graminearum*** **and (ii) chitosan +** ***F. graminearum***							
M415T410_2	6.84	415.1367	416.1440	1.61 1.79	1-Acetoxypinoresinol* Aliarin 4'-Methylliquiritigenin 7-rhamnoside 3-Hydroxy-1-[(4-methoxy-7-oxo-7H-furo[3,2-g]chromen-9-yl)oxy]-3-methyl-2-Butanyl (2E)-2-methyl-2-butenoate?	416.1471 - - -	7 - - -
M225T568	9.49	225.1125	226.1198	1.99 2.08	Epi-4'-hydroxyjasmonic acid* 12-Hydroxyjasmonic acid* Allixin	226.1205 - -	3 - -
M679T849	14.14	679.374	680.3813	1.77 1.67	Dianversicoside C	680.3772	6
M227T1549	25.81	227.1287	228.1360	4.95 5.01	Traumatic acid (-)-11-Hydroxy-9,10-dihydrojasmonic acid* (-)-12-Hydroxy-9,10-dihydrojasmonic acid* 9,12-Dioxo-dodecanoic acid	228.1362 - - -	0.9 - - -
M209T1549	25.83	209.1179	210.1252	2.53 2.40	Jasmonic acid Iso-jasmonic acid (R)-8-Acetoxycarvotanacetone	210.1256 - -	2 1 -
**Downregulated by both (i) chitosan and (ii)** ***F. graminearum***							
M283T608	10.17	283.0857	284.0930	0.53 0.38	6-Formylindolo [3,2-B] carbazole	284.0950	7
**Downregulated by both (i) chitosan and (ii) chitosan +** ***F. graminearum***							
M345T446	7.51	345.0640	346.0713	0.59 0.57	Tetrahydroxy-dimethoxyflavone	346.0689	6

The exact *m/z* value is the monoisotopic mass of the compound suggested for annotation. The error is the relative difference between the experimental neutral *m/z* value and the exact *m/z*. The asterisk indicates the metabolite that most likely corresponds to the feature

*RT* retention time, *FC* fold change

^a^ Features were annotated based on METLIN and literature review

### Identification of the metabolites regulated by either chitosan or *F. graminearum*

A total of 51 features were differentially produced in wheat spikes response to chitosan and not in response to *F. graminearum* (Fig. 4). Two of these were up-regulated but not annotated while 49 were down-regulated by chitosan of which one (M395T189) was putatively identified as either apigenin triacetate (3 ppm error) or 3,5,6-trimethoxy-3',4'-methylene-dioxyfurano[2,3:7,8] flavone (Table 1).

In total, 32 features were potentially differentially regulated in response to *F. graminearum* but not in response to chitosan (Fig. 4). Among the 21 features potentially up-regulated by *F. graminearum*, 3 were putatively annotated (Table 1). The feature M293T380 was putatively identified as distichonic acid (2 ppm error). A second feature (M365T570) was putatively identified as a glucoside; either 7-methyl-3-methylene-1,2,6,7-octanetetrol-2-glucoside or p-menthane-1,2,8,9-tetrol-9-glucoside (4 ppm error for both). The third metabolite (M327T1531) corresponded to several entries in METLIN (2 ppm error for all of them) that can be sub-classified into two categories: trihydroxyoctadecadienoic acids (TriHODE) and hydroperoxyl-epoxy-octadecenoic acid. Among the 11 features potentially down-regulated by *F. graminearum*, 2 were putatively annotated (Table 1). One feature (M310T230) was identified as cinnamoyl β-D-glucoside (4 ppm error) and the other (M403T278) as mollicellin D (4 ppm error). Mollicellin D is a mycotoxin produced by the fungus *Chaetomomium brasiliense* [18] which is a plant endophyte and there has been no previous report of this toxin being detected in wheat spikes.

### Identification of the metabolites primed by chitosan to respond to *F. graminearum*

Metabolites differentially responsive to both agents, but not to either agent on its own, were delineated. In total, 117 features were differentially produced in response to the combination of chitosan and *F. graminearum* (Fig. 4) and the putatively annotated features are denoted in Table 1. Five of the 66 features potentially up-regulated by the combined treatment were putatively annotated. The feature M497T463 was identified as iridodial glucoside tetraacetate or 8-epiiridodial glucoside (4 ppm error), both of which are involved in the same metabolic pathway [19], M595T1405 was putatively identified as salannin (7 ppm error), and M663T1079 matched three plant metabolites in METLIN: phytolaccoside B, medicagenic acid, 3-O-beta-D-glucoside and elatoside G (4 ppm error). Two features (M565T1500 and M565T1532) were both identified as 25-cinnamoyl-vulgaroside (8 and 7 ppm error respectively) [20]. Since their retention times and *m/z* values are very similar, it is highly likely that these features correspond to the same compound. Among the 51 features potentially down-regulated by chitosan and *F. graminearum* together, 2 were putatively annotated (Table 1). One feature (M434T773) was identified as cadabicine (0.7 ppm error) and the second one (M469T1211) corresponded to four oxygenated terpenoids (6 ppm error). However, it was not possible to determine the exact identity of the metabolite.

### Identification of the metabolites responsive to both chitosan and *F. graminearum*

Three features were found to be regulated by both chitosan and *F. graminearum* (1 was up-regulated and 2 were down-regulated). A total of 24 features were found to be regulated by chitosan alone and in combination with fungus (chitosan + *F. graminearum*): 13 were up-regulated and 11 were down-regulated. Seven features were regulated by fungus alone or in combination with chitosan (in *F. graminearum* and chitosan + *F. graminearum* treatments): 6 were up-regulated and 1 was down-regulated). Finally, only 1 feature was found to be commonly regulated by all three treatments, being up-regulated in response to chitosan, *F. graminearum* and chitosan + *F. graminearum* compared to mock treatment (Table 2).

The only feature up-regulated by both chitosan and *F. graminearum* could not be identified by searching in METLIN or into the literature. One feature down-regulated by chitosan and *F. graminearum* (M283T608) was potentially identified as 6-formylindolo [3,2-b] carbazole (FICZ; Table 2). It is a degradation product of tryptophan under exposure to visible light [21]. However, it has been found in humans but not in plants thus far.

Of the 13 features potentially up-regulated by chitosan and chitosan + *F. graminearum*, 3 were putatively annotated (Table 2). One feature (M218T324) was identified as nigellimine N-oxide, alpha-hydroxy-1-methyl-1H-indole-3-propanoic acid or alpha-methoxy-1H-indole-3-propanoic acid (3 ppm error). However, none of these compounds were reported to be related to cereals in previous studies. The third putatively annotated feature (M687T1403) up-regulated by chitosan and chitosan + *F. graminearum* was identified as callichiline (4 ppm error), which is a metabolite found in various plant families, but not in cereals [22].

All of the features found to be potentially upregulated by *F. graminearum* and chitosan + *F. graminearum* corresponded to at least one entry in METLIN (Table 2). One feature (M415T410) matched four metabolites in METLIN: aliarin, 1-acetoxypinoresinol, 4'-methylliquiritigenin 7-rhamnoside and 3-hydroxy-1-[(4-methoxy-7-oxo-7H-furo[3,2-g]chromen-9-yl)oxy]-3-methyl-2-butanyl (2E)-2-methyl-2-butenoate (7 ppm error). These compounds were all already isolated from plants apart from 3-hydroxy-1-[(4-methoxy-7-oxo-7H-furo[3,2-g]chromen-9-yl)oxy]-3-methyl-2-butanyl-(2E)-2-methyl-2-butenoate. The molecule 1-acetoxypinoresinol seems to be the most likely to correspond to the feature since it is a phenolic that was found to be potentially involved in FHB resistance [23–25]. It is a lignan found in olive oil in high quantities [26]. One feature (M679T849) upregulated by *F. graminearum* and chitosan + *F. graminearum* was putatively identified as dianversicoside C (6 ppm error). It is a triterpenoid saponin found in the herb *Dianthus versicolor* [27], used in Chinese medicine, but no study reported it in cereals. Finally, several features upregulated by *F. graminearum* and chitosan + *F. graminearum* were found to potentially correspond to metabolites involved in the jasmonic acid pathway. A feature (M225T568) was identified as epi-4'-hydroxyjasmonic acid, 12-hydroxyjasmonic acid or allixin (3 ppm error). The two hydroxyjasmonic acids are involved in the metabolism of jasmonic acid and allixin is a phytoalexin found in garlic [28]. It is therefore more likely that the extracted metabolite corresponds to an hydroxyjasmonic acid rather than to allixin. Two other features up-regulated by both *F. graminearum* and chitosan + *F. graminearum* are metabolites potentially involved in the jasmonic acid pathway, but they also matched with other compounds in METLIN. One metabolite (M227T1549) was putatively identified as traumatic acid, (-)-11-hydroxy-9,10-dihydrojasmonic acid, (-)-12-hydroxy-9,10-dihydrojasmonic acid or 9,12-dioxo-dodecanoic acid. Traumatic acid is a cytokinin found in plants and is a wound healing compound that stimulates cell division and forms a callus [29, 30]. The metabolite 9,12-dioxo-dodecanoic acid is a fatty acid which derives from lauric acid (dodecanoic acid) found in high concentrations in coconut and palm kernel oils. It is therefore more likely that the unknown metabolite is traumatic acid or hydroxy-9,10-dihydrojasmonic acid. The last feature (M209T1549) upregulated by *F. graminearum* and chitosan + *F. graminearum* was putatively identified as jasmonic acid, iso-jasmonic acid or (R)-8-acetoxycarvotanacetone (2 ppm error for jasmonic acid and 1 ppm error for the other compounds). The molecule (R)-8-acetoxycarvotanacetone is found in cardamom (*Elettaria cardamomum*) oil [31]. It is therefore more likely that the feature corresponds to jasmonic or iso-jasmonic acid than to (R)-8-acetoxycarvotanacetone. The metabolite downregulated by *F. graminearum* and chitosan + *F. graminearum* could not be identified.

Only one feature was found to be potentially regulated by chitosan, *F. graminearum*, and chitosan + *F. graminearum* and it was downregulated compared with the samples treated with water and inoculated with Tween 20. However, the m/z value of the feature did not match any compound in METLIN or in the literature (Table 2).

## Discussion

The antifungal activity tests determined that chitosan could inhibit the growth of *F. graminearum* GZ3639 under *in vitro* conditions. Similar antifungal activity tests conducted on PDA had already shown that chitosan could inhibit the growth of *F. graminearum* [10, 11] and *F. culmorum* [5]. The differences observed here between the experiments done with PDA and PDB could be caused by the different growth stages of the fungus at the time of inoculation of the media. Indeed, the PDA medium was inoculated with a mycelial plug and the hyphal growth was measured [32], whereas the PDB medium was inoculated with macroconidia and their germination and proliferation were measured over time. Based on the results, one can surmise that chitosan has a stronger activity against spore germination than hyphal development. Other antifungal activity tests could be performed to validate this, including the microscopic assessment of the effects on spore germination and elongation and hyphal growth and the use of staining techniques to observe the effect of the products on the fungal health.

The FHB experiment conducted under glasshouse conditions determined that spraying the spikes of the wheat cv. Remus (susceptible to FHB) with chitosan decreased the disease severity when it was applied pre- or post-inoculation with *F. graminearum.* However, chitosan significantly reduced the negative effects of FHB on grain development (grain weight per head, number of grains per head and 1000-grain weight) only when it was applied 24 h before, but not after *F. graminearum* inoculation. Therefore, its ability to reduce the disease severity was more durable when applied as a pre- rather than a post-inoculant. Previous studies have demonstrated the efficacy of chitosan against FHB when applied 24 h pre-inoculation with *F. graminearum* [9], just before inoculation [10] and after inoculation [11], and 3 or 5 days post-inoculation [10]. As stated in the introduction, chitosan was found to be more effective at reducing the disease severity when applied as a pre-inoculation treatment rather than when applied 3 or 5 days post-inoculation [10]. This concurs with the findings herein where chitosan applied as a pre-inoculant reduced the grain yield loss caused by FHB compared with controls (water) but not when applied as a post-inoculant. The fact that treating wheat heads with chitosan post-inoculation did not significantly decrease yield loss could be explained by the very high disease severity obtained by leaving the bags on the heads for 4 days instead of 2 days in most studies. This confirms that it is critical to treat the plants before infection to effectively mitigate the effects of severe disease. It had been shown that the infection of spikelets by *F. graminearum* was slower when the spikes had been treated with chitosan may be due to the accumulation of hydrogen peroxide in the spikelets arising from exposure to chitosan [10, 11]. This suggests that chitosan exhibits not only antifungal properties, but is also able to activate host defence responses. According to the literature, it is possible that chitosan creates a physical barrier between the wheat spikes and *F. graminearum* by agglutination around the penetration sites, stimulating plant defence mechanisms by triggering a hypersensitive reaction around the agglutination sites and the accumulation of reactive oxygen species and pathogenesis related (PR) proteins [4, 6, 17]. PR proteins can have several effects that slow down the infection process, such as reinforcing the lignification and inducing the accumulation of phenolic compounds with antifungal activity. The effects of chitosan observed here may be two-fold: (i) antifungal activity (that was observed during *in vitro* experiments), and (ii) stimulation of plant defence mechanisms before or during the infection process. Herein, it was decided to perform an untargeted metabolomics study to investigate the effect of chitosan used as a pre-inoculant on the production of secondary metabolites in wheat and against FHB.

This was the first untargeted metabolomics study conducted to investigate the effect of chitosan on the metabolite profile of wheat spikes. It was previously determined that spraying wheat seedlings with chitosan stimulated the carbon and nitrogen metabolism in the leaves [13]. Other studies investigated the impact of chitosan on the metabolome of the medicinal plant *Hypericum perforatum* L. [33], white clover (*Trifolium repens*) [34], grapes (*Vitis vinifera* L.) [35] and the cells of *Nicotiana tabacum* [36]. They showed that chitosan stimulated the accumulation of a variety of compounds such as amino acids, sugars, organic acids and flavonoids. Metabolomics studies had been performed to get an insight into the defence mechanisms of wheat and barley against infection by *F. graminearum* [14, 15, 20, 37–39] and production of DON by *F. culmorum* [40]. These studies reported that the metabolites related to plant resistance under these conditions were mainly linked to the metabolism of phenylpropanoid, flavonoids, fatty acids and terpenoids. Apart from one study [14] which was performed on samples harvested 24 h post-inoculation (hpi), all others were conducted with samples harvested at least 48 hpi. Proteomics studies have shown that the proteome of the plants was modified as soon as 6 hpi with *F. graminearum* [41]. The aim of this research was to investigate the resistance-related mechanisms occurring early during the infection process (6 and 24 hpi).

The result of the putative identification of one feature down-regulated by chitosan was surprising. It was likely to correspond to apigenin triacetate. Apigenin triacetate derives from apigenin, a flavonoid that was previously found to be potentially involved in wheat resistance against FHB and derives from naringenin in the phenylpropanoid pathway [19, 23]. This pathway was also shown to be up-regulated in wheat after inoculation with *F. culmorum* but not when the plants had been treated with fungicides [42]. The phenylpropanoid pathway is involved in the production of antimicrobial compounds and cell wall reinforcement by lignification, which can prevent the infection by the pathogen [37]. A study showed that apigenin applied on wheat spikes pre-inoculation with *F. graminearum* spores increased their resistance to infection by the fungus [43]. Another study showed that incubating *F. graminearum* and *F. culmorum* in the presence of apigenin can reduce or increase the production of trichothecenes *in vitro* [44]. Apigenin was also found to be involved in the resistance of other plants to infection by fungal pathogens. It is one of the most abundant flavonoids in soybean and has been shown to inhibit the growth of soybean fungal pathogens (e.g. *Colletotrichum truncatum* and *Rhizoctonia solani*) *in vitro* [45]. It was also found to accumulate in sorghum seedlings after inoculation with *Colletotrichum sublineolum*, which causes the disease anthracnose, and to inhibit spore germination of the fungus *in vitro* [46]. However, even though several metabolites derived from apigenin were previously identified in wheat, apigenin triacetate has not been reported in plants and the metabolic reaction that links it to apigenin has not been established. Since the phenylpropanoid pathway had been found to be up-regulated in wheat by *F. culmorum*, its potential down-regulation by chitosan in this study suggests that chitosan may trigger different defence reactions in wheat as compared to FHB [42].

Among the features that were up-regulated by *F. graminearum*, was the phytosiderophore distichonic acid which has been shown to be excreted by plant roots under iron deficiency [47]. They participate in increasing the uptake of iron by the plants by forming a complex with Fe^3+^ ions contained in the soil and in distributing it through the plants. Siderophores were found in barley leaves [48] but there is no evidence to date of the presence of siderophores in cereals spikes. Another feature would need to be confirmed using a chemical standard as it matched two interesting metabolites in METLIN: TriHODE which was found to be potentially involved in wheat resistance against FHB [23, 49] and 9-hydroperoxy-12,13-epoxy-10-octadecenoic acid. Among the metabolites down-regulated during infection was identified as cinnamoyl beta-D-glucoside which is derived from a trans-cinnamic acid reacting with a beta-D-glucose. Cinnamic acid had previously been found in higher concentrations in wheat and barley cultivars resistant to FHB than in susceptible ones [20, 37, 38]. Cinnamic acids are precursors in the production of lignans, which are compounds reinforcing plant cell walls, preventing infection by *Fusarium*. However, it was also proven that cinnamic acid stimulated the accumulation of DON in wheat heads [40]. In our study with a susceptible wheat cultivar, it appears that *F. graminearum* down-regulated the production of cinnamic acids and this may have prevented the stimulation of lignification as a defence mechanism.

This metabolomic study also showed that more features were regulated when both chitosan and the inoculum of *F. graminearum* spores were sprayed on the wheat spikes as compared with chitosan or *F. graminearum* on their own (117 vs 51 and 32, respectively, Fig. 4). Hence, these potential metabolites are primed by chitosan to respond to infection by *F. graminearum*. Three features up-regulated by chitosan and *F. graminearum* together were putatively identified as compounds that were already found to be potentially involved in wheat or barley resistance against FHB: iridodial glucoside tetraacetate or 8-epiiridodial glucoside (same metabolic pathway), salannin and 25-cinnamoyl vulgaroside [19, 20, 23]. The potential role(s) of the terpene iridodial glucoside tetraacetate and the terpenoid 25-O-cinnamoyl vulgaroside in plant metabolism have not been established. However, the liminoid salannin is known for its anti-feedant and insecticidal activities [50]. Studies have also shown that salannin inhibited the growth of the fungal phytopathogens *Drechslera oryzae* and *Fusarium oxysporum* f. sp. *vasinfectum* [51]. Therefore, this compound might play a role in reducing wheat infection by *F. graminearum* by directly inhibiting the fungal growth. Regarding the metabolites that were down-regulated by the combination of chitosan and *F. graminearum*, one was identified as cadabicine which is unexpected as it was previously reported to be up-regulated in resistant wheat cultivar under infection by *F. graminearum* [49]. However, its role was not discussed. Cadabicine is an alkaloid found in high concentration in plants of the genus *Cadaba* [52], and extracts of *Cadaba farinosa* have been shown to inhibit the mycelial growth of *F. oxysporum* f. species *in vitro*.

The metabolomic study indicated that the jasmonic acid pathway was upregulated by *F*. *graminearum* alone and chitosan + *F. graminearum*. Jasmonic acid and its derivatives hydroxyjasmonic acid and hydroxy-9,10-dihydrojasmonic acid were found in higher concentrations in spikes that had been inoculated with fungus, as compared to those that were not, irrespective of chitosan treatment. This is consistent with previous studies demonstrating that the jasmonic acid pathway plays a role in wheat resistance against *Fusarium* [19, 20, 38, 39]. Metabolites of the jasmonic acid pathway are known to induce the expression of defence-related genes in plants under stress conditions, especially plants infected by necrotrophic pathogens [53]. Jasmonic acid also directly inhibits the growth of *F. graminearum* [54, 55]. It can also stimulate the production of phytoalexins, which are compounds having direct antimicrobial activities.

The metabolites 6-formylindolo [3,2-B] carbazole (FICZ) and a tetrahydroxy-dimethoxyflavone were potentially downregulated by chitosan and *F. graminearum* and by chitosan and chitosan + *F. graminearum* respectively*.* FICZ derives from tryptophan under exposure to visible light (only observed in humans so far) [21]. Tryptophan is part of the auxin pathway in plants, where it is converted into indole-3-pyruvate, which is then converted into indole-3-acetic acid (IAA )[56]. *F. graminearum* can also produce IAA in the early stage of infection of wheat heads, resulting in the accumulation of the hormone in the heads [57]. IAA in wheat infected by *F. graminearum* was shown to be associated with susceptibility to the fungal infection [58, 59]. Additionally, *in vitro* experiments showed that exogenous IAA significantly increased the production of the mycotoxin 15-acetyldeoxynivalenol (ADON), even though it also inhibited the mycelial growth of *F. graminearum* [60]. Tetrahydroxy -dimethoxyflavones have previously been found to be potentially involved in resistance against FHB [23]; in this study the accumulation of this feature was downregulated by chitosan (with or without the pathogen) and the role of these flavanones remains unknown.

## Conclusions

In conclusion, this research showed that chitosan inhibited the spore germination and hyphal development of *F. graminearum in vitro*. Additionally, chitosan decreased FHB disease severity when applied as a spray inoculant on the spikes of the susceptible wheat cv. Remus, either pre- or post-fungal inoculation. However, it only reduced the yield loss caused by the infection when applied as a pre-inoculant. The untargeted metabolomic study determined that chitosan applied as a pre-inoculant had an impact on the metabolites within the wheat spikes, whether the plants had been inoculated with *F. graminearum* spores or not. Some of the putatively identified metabolites were consistent with previously published studies whereas several metabolites signatures of interest remain to be characterized. Validation studies are needed to confirm the pathways delineated in this study, and particularly the uncharacterized metabolites, and *in vitro* and *in vivo* experiments are needed to study the impact of metabolites of interest on the infection of plants by pathogens. Targeted measurements with higher throughput analytical methods and the use of reference compounds may help elucidate the defence mechanisms of plants growing under biotic stress conditions and also to validate the identification of all potential depicted metabolites. Finally, in agreement with previous studies, chitosan was proven to significantly reduce the impact of FHB in wheat, suggesting that a treatment could be optimised for field application. Further studies would be necessary to compare the effect of different types of chitosan and to determine an optimal time of application regarding infection.

## Methods

### Chitosan and fungal material

Water-soluble chitosan hydrochloride (CAS number 9012-76-4, molecular formula (C_6_H_11_NO_4_)_n_, molecular weight 195 kDa, degree of deacetylation 90%, viscosity 10-50 cps, food grade) was supplied by Shandong Laizhou Highly Bio-products Co. Ltd. (China). *F. graminearum* strain GZ3639 was provided by Dr Robert Proctor (USDA-ARS-NCAUR, Peoria, USA). It was first isolated from wheat in a field in the USA [61]. It was stored long term in sterile 10% glycerol solution (v v^-1^; Fisher Scientific, United Kingdom) at -70 °C. The fungus was cultured on potato dextrose agar (Oxoid, United Kingdom) at 21 °C for 7 days prior to use. Conidia of *F. graminearum* GZ639 were produced in mung bean broth (MBB; Tesco, United Kingdom) [62]. Conidia were rinsed several times with sterile distilled water and collected by centrifugation for 20 min. at 4,000 rpm. They were resuspended in sterile distilled water and their concentration was determined using a haemocytometer (Kova International, USA), and adjusted to a concentration of 10^5^ conidia mL^-1^ with sterile distilled water for the

the Fusarium head blight experiments. The experiments were performed with fresh conidial solutions.

### Plant material

Seeds of the spring common wheat (*Triticum aestivum*) cultivar (cv.) Remus were originally provided by Prof. Hermann Buerstmayr (Institute of Plant Breeding and Institute of Biotechnology in Plant Production, University of Natural Resources and Life Sciences, Vienna, Tulln, Austria). This cultivar is susceptible to infection by *F. graminearum* [63]. This cultivar can be obtained from the John Innes Germplasm Resource Unit (idPlant: 16594, GRU Store Code: W3173). Seed and were bulked and maintained in UCD; plants were propagated under greenhouse conditions in UCD Rosemount Environmental Research Station (Dublin, Ireland) at 20-24 °C with a 16/8 h light/dark photoperiod at 300 μmol m^-2^ s^-1^ and 70% relative humidity. All methods, including plant experimental research, were performed in accordance with the relevant guidelines, regulations and legislation.

### *In vitro* antifungal activity tests

Two *in vitro* antifungal activity tests were performed to study the dose response effect of chitosan on the growth of *F. graminearum* on solid PDA and in liquid PDB (Scharlab, Spain). For the solid culture tests, chitosan was incorporated into sterile PDA (Scharlab, Spain) to a final concentration of 0.0, 0.1, 0.125, 0.15, 0.175 or 0.2% (w v^-1^). The plates were centrally inoculated with a 4-mm PDA plug of *F. graminearum* GZ3639 with consistent contact and incubated in the dark at 21 °C for 6 days. For each plate, the mycelial growth diameter was measured at day 2, 4 and 6. Also, at day 6, the percentage of growth inhibition by chitosan, relative to mock treatment, was calculated according to the formula: percentage inhibition = (DC – DT) x 100 / DC, where DC = mycelial diameter (mm) of the control and DT = mycelial diameter (mm) for a given treatment. The data presented for the solid culture experiment represent the results obtained for 12 plates per treatment (from three replica trials, each including four plates per concentration tested).

Lower chitosan concentrations were used for the liquid culture as compared to the solid culture experiment because preliminary experiments elucidated that fungal growth in PDB was more sensitive to chitosan, as compared to that in solid PDA medium. PDB medium (Scharlab, Spain) was gently mixed with chitosan to obtain a final concentration of 0.0, 0.00675, 0.0125, 0.025, 0.05, 0.1 or 0.2% (w v^-1^). Liquid culture antifungal tests were performed in 96-well microtitre plates: wells contained 180 μL of amended PDB medium and 20 μL of either *F. graminearum* conidia (to obtain a final concentration of 10^5^ conidia mL^-1^) or sterile water (control). Plates were incubated in a shaker at 21 °C at 150 rpm in the dark for 4 days. The optical density at 600 nm [64–66] was measured every 24 hours for 4 days using a spectrometer (SPECTROStar Nano, BMG LABTECH, Germany) and the percentage of growth inhibition (growth relative to control) was assessed at day 4 according to the formula: percentage inhibition = (ODC – ODT) x 100 / ODC, where ODC = optical density of the control and ODT = optical density with the treatment. The data presented for the liquid culture experiment represent the results obtained for 12 flasks per treatment (from three replica trials, each including four wells per concentration tested).

Statistical analyses of the *in vitro* antifungal activity test data was performed using the software IBM SPSS Statistics 24 (International Business Machines Corporation, USA). The normality of the data sets was assessed based on the Shapiro-Wilk test because the sample sizes were inferior to 50. The data sets followed a normal distribution. The analyses were performed using one-way ANOVA test with a Games-Howell post-hoc analysis because Levene’s test for the homogeneity of variance had failed. The data were represented with line charts.

### Fusarium head blight experiment for phenotyping and yield analysis

Two FHB experiments were conducted at the same time to determine the impact of chitosan on disease development and associated yield losses whether it is applied to wheat heads pre- or post-fungal inoculation. Data presented for both experiments represent the values (and averages) obtained for five replica trials for disease assessment and three for yield analysis, each trial including at least twenty heads per treatment combination. Table S1 details the total number of heads used for disease and yield component assessments in both experiments.

Seeds of the wheat cv. Remus were surface-sterilized for 10 min. in 1% sodium hypochlorite solution (v v^-1^; VWR International Ltd., France), rinsed three times with sterile distilled water, and air-dried on Whatman grade 1 filter paper [67]. The seeds were germinated on filter paper moistened with 5 mL of sterile distilled water at 21 °C in the dark for 4 days. The seedlings were then transferred to 3 L pots filled with John Innes Compost No. 2, two seedlings per pot, and grown under greenhouse conditions at UCD Rosemount Environmental Research Station (Dublin, Ireland) at 20-24 °C with a 16/8 h light/dark photoperiod at 300 μmol m^-2^ s^-1^ and 70% relative humidity. At mid-anthesis (growth stage 65 [68]), the heads of the two first secondary tillers of each plant were treated 24 hours pre- or post-fungal inoculation as described below.

For treatment pre-inoculation, the heads were treated by spraying with 2 mL of either water (mock as a control) or 0.2% chitosan (w v^-1^) and were spray-inoculated 24 hours later with 2 mL of either 0.02% Tween 20 as a mock or *F. graminearum* conidia (10^5^ conidia mL^-1^ in 0.02% Tween 20). The heads were then covered with plastic bags (Pro-loc, Sparks lab supplies, Ireland) for 4 days to increase humidity and thus promote fungal infection. For treatment post-inoculation, the heads were first spray-inoculated with 2 mL of either 0.02% Tween 20 as a mock or *F. graminearum* conidia (10^5^ conidia mL^-1^ in 0.02% Tween 20), covered with plastic bags, and treated 24 hours later with 2 mL of either water (mock as a control) or 0.2% chitosan (w v^-1^). The bags were also kept for 4 days post-inoculation in total to promote fungal infection. The percentage of infected spikelets (bleached or with brown/black lesions) per head was assessed at days 7, 14 and 21 post-inoculation and the AUDPC was calculated [69]. The yield component analysis was performed by measuring the average grain weight per head (mg), the number of grains per head and the 1000-grain weight (g) on a per head basis.

Statistical analyses of the disease and yield component data were performed using the software IBM SPSS Statistics 24. The normality of the data sets was assessed based on the Kolmogorov-Smirnov test because the samples sizes were superior to 50. The data sets were analysed using the Kruskal-Wallis test and were represented as box plots because they did not follow a normal distribution.

### Fusarium head blight experiment for metabolomic analysis

The second FHB experiment was conducted to determine the impact of chitosan on the metabolome of wheat spikes in the presence and absence of *F. graminearum.* The plant (wheat cv. Remus) growth conditions were as described above for the FHB experiments. At mid-anthesis (growth stage 65) the heads of the two first secondary tillers were sprayed with 2 mL of either water or 0.2% chitosan (w v^-1^) and spray-inoculated 24 hours later with 2 mL of either 0.02% Tween 20 or *F. graminearum* conidia (10^5^ conidia mL^-1^ in 0.02% Tween 20). Spikes were covered with a plastic bag and harvested at either 6 or 24 hours post-fungal inoculation. Spikes were flash frozen in liquid nitrogen and stored at -80°C prior to metabolite extractions. The experiment comprised three replica trials, each including six spikes per experimental group (treatment x inoculation x harvest time), which were subsequently pooled to yield one bulk sample per group).

### Untargeted UHPLC-QTOF-MS metabolite analysis

Flash frozen spikes were freeze-dried overnight, ground to a fine powder in liquid nitrogen using sterile mortars and pestles and homogenised in a tissue lyser (high speed shaker to disrupt biological materials, Qiagen, Netherlands). For each sample, 200 ± 0.5 mg of ground tissue were extracted in 5 mL cold 70% aqueous methanol (v v^-1^; hypergrade for LC-MS LiChrosolv®, Merck, Ireland) acidified with 0.1% formic acid (v v^-1^; Sigma-Aldrich, USA) and containing 62.4 μL of a 11 mM methyl vanillate solution (Sigma-Aldrich, USA) as the internal standard. The samples were vortexed and sonicated for 20 min. at 0 °C. The homogenates were then centrifuged at 3,000 g for 10 min at 4 °C and the supernatants were filtered using a 0.22 μm PVDF syringe filter (Millipore, Merck, Ireland). The methanolic content of the extract was dried under a nitrogen stream at 0 °C for 3.5 hours and the aqueous part was then freeze-dried overnight. The resulting extracts were stored at -20 °C. A quality control sample was obtained by performing the extraction of the mix of 10 samples of 20 mg each across the three replica trials. Blanks were also prepared for each replicate. The extracts were finally reconstituted in 500 μL of 70% methanol solution (v v^-1^) containing 0.1% formic acid (v v^-1^) and analysed by ultra-high performance liquid chromatography quadrupole time-of-flight mass spectrometer (UHPLC-QTOF-MS).

Chromatographic runs were conducted on a UHPLC system (1290 Infinity II LC System, Agilent, Ireland) using a reverse phase column (ZORBAX RRHD Eclipse Plus C18, 2.1 × 50 mm, 1.8 μm, Agilent, Ireland) with a flow rate of 200 μL min^-1^. The injection volume of samples was 2 μL and the extracts were randomised and analysed in a single batch experiment. The mobile phase was nanopure water, containing 0.1% formic acid (v v^-1^) and acetonitrile and the following elution gradient was used: 0-2 min.: 5% acetonitrile; 2-27 min.: 5-35% acetonitrile; 27-34 min.: 35-95% acetonitrile and 34-35 min.: 5% acetonitrile (v v^-1^). The mass spectral analyses were conducted with a high resolution quadrupole/time of flight mass spectrometer (6550 iFunnel QTOF LC/MS, Agilent, Ireland) equipped with an electrospray ionization (ESI) source (Dual AJS ESI, Agilent, Ireland) in negative-mode (dry gas flow of 12 L min^-1^ at 190 °C, -3.5 kV capillary voltage, 35 psi nebulizer, 5 V collision energy and 1.5 kV nozzle voltage) which was operated in 100-1,400 *m/z* mass range. For internal mass calibration during the MS analysis, reference masses of TFA (*m/z* 112.9856), Purine (*m/z* 119.0363), HP-0285 (*m/z* 301.9981, HP-0285 + OH adduct), HP-0921 (*m/z* 966.0007, HP-0921 + formate adduct and *m/z* 1033.9881, HP0921) were used.

### Metabolite data processing and statistical analysis

The UHPLC-QTOF-MS data were processed using the web-based platform Galaxy Workflow4Metabolomics [70]. The statistical analyses between two categories of samples were performed with the online software MetaboAnalyst (https://www.metaboanalyst.ca/). Four categories of samples were considered for the analysis: water treatment and Tween 20 inoculation (W_T), water treatment and *F. graminearum* inoculation (W_F), chitosan treatment and Tween 20 inoculation (C_T) and chitosan treatment and *F. graminearum* inoculation (C_F). The samples corresponding to the 6- and 24-h time points were pooled together which resulted in a total of 6 replicas per category. Three category comparisons were performed: W_T vs C_T to determine the metabolites regulated by chitosan, W_T vs W_F to determine the metabolites regulated by *F. graminearum* and W_T vs C_F to determine the metabolites regulated when both chitosan and *F. graminearum* had been applied to the plants. Volcano plots delineated the features of interest (potential metabolites that were differentially produced between two sample categories): i.e. those with a statistically significant fold change (FC) of at least 1.5 (t-test: *P* ≤ 0.05).

### Metabolite annotation

Before annotating the features of interest described above, their calculated retention time and *m/z* value were compared with the experimental values of the main peaks found on the spectra extracted from the extracted ion chromatograms (EIC), which were obtained from the total ion chromatograms (TIC) of the samples. Only the features of interests that corresponded to peaks in terms of retention time and *m/z* value were kept for the annotation. This was performed using the molecular feature extraction algorithm of the MassHunter Workstation software – Qualitative analysis (Agilent, USA). The features were annotated using the online databases METLIN (metabolite database), KNApSAcK (plant species-metabolite relationship database [71]), HMDB (Human Metabolome Database) and PubChem (chemical database) and using published data [15, 20, 72–75]. A maximum error of 10 ppm was allowed between the neutral measured mass and the monoisotopic exact *m/z* values.

## Supplementary Information

**Additional file 1: Figure S1.** *In vitro* growth inhibition of *F. graminearum* GZ3639 by chitosan. (A) Potato Dextrose Agar (PDA) was prepared with chitosan to final concentrations of 0.0, 0.1, 0.125, 0.15, 0.175 or 0.2% (w v^-1^) and was inoculated with a plug of *F. graminearum* GZ3639. The percentage of growth inhibition was calculated after 6 days of incubation based on the mycelial growth diameter measured. (B) Potato Dextrose Broth (PDB) was prepared with chitosan to final concentrations of 0.0, 0.00675, 0.0125, 0.025, 0.05, 0.1 or 0.2% (w v^-1^) and was inoculated with spores of *F. graminearum*. The percentage of growth inhibition was calculated after 4 days of incubation based on optical density measured. Error bars represent the standard error of the means. Asterisks above the data sets indicate that the data are statistically significantly different from the mock water treatment, according to a one-way ANOVA test (* = *P* ≤ 0.05, ** = *P* ≤ 0.01, *** = *P* ≤ 0.001).

**Additional file 2: Figure S2.** Box plot distribution of the effect of spraying wheat heads with chitosan on the percentage of spikelets infected with *F. graminearum*. The heads of wheat cv. Remus were sprayed at mid-anthesis with water (control) or 0.2% chitosan (w v^-1^) and were spray-inoculated 24 hours before (A) or after (B) with 0.02% Tween 20 (mock) or 10^5^ spores ml^-1^ *F. graminearum* GZ3639. Asterisks above the data sets indicate that the data are statistically significantly different from the mock water treatment, according to Kruskal-Wallis tests (*** = *P* ≤ 0.001).

**Additional file 3: Figure S3.** Representation of the Volcano plots delineating the features of interest. The features of interest were selected based on the fold change (FC, cut-off value 1.5) and the *P* value of the t-tests (*P* ≤ 0.05) performed to compare two sample categories. The samples corresponding to the 6- and 24-hour time points were pooled together for the statistical analysis. Three comparisons were performed: (A) chitosan (samples W_T compared with C_T, features regulated by chitosan), (B) *F. graminearum* (samples W_T compared with W_F, features regulated by *F. graminearum*) and (C) chitosan + *F. graminearum* (samples W_T compared with C_F, features regulated by the combination of chitosan and *F. graminearum*). The graphs represent the features that were significantly up- (red) or down-regulated (blue) or not selected for subsequent analysis (grey).

**Additional file 4: Table S1.** Total number of heads analysed (disease and yield component analysis) across the three replicate Fusarium head blight trials.

## Data Availability

All data and materials are included in the article and the supplemental files.

## Abbreviations

ADON: Acetyldeoxynivalenol
AUDPC: Area under disease progress curve
DON: Deoxynivalenol
Dpi: Day post-inoculation
EIC: Extracted ion chromatograms
ESI: Electrospray ionisation source
FC: Fold change
FHB: Fusarium head blight
FSB: Fusarium seedling blight
FRR: Fusarium root rot
Hpi: Hours post-inoculation
IAA: Indole-3-acetic acid
MBB: Mung bean broth
PDA: Potato dextrose agar
PDB: Potato dextrose broth
TIC: Total ion chromatograms
TriHODE: 9S-Hydroxy-10E,12Z-octadecadienoic acid
U-HPLC-QTOF-MS: Ultra-high performance liquid chromatography quadrupole time-of-flight mass spectrometer
