# Supplementary figures and images for: The impact of chitosan on the early metabolomic response of wheat to infection by Fusarium graminearum
Source: BMC Plant Biol. 2022 Feb 19;22:73. doi: 10.1186/s12870-022-03451-w (PMC8857839; doi:10.1186/s12870-022-03451-w)

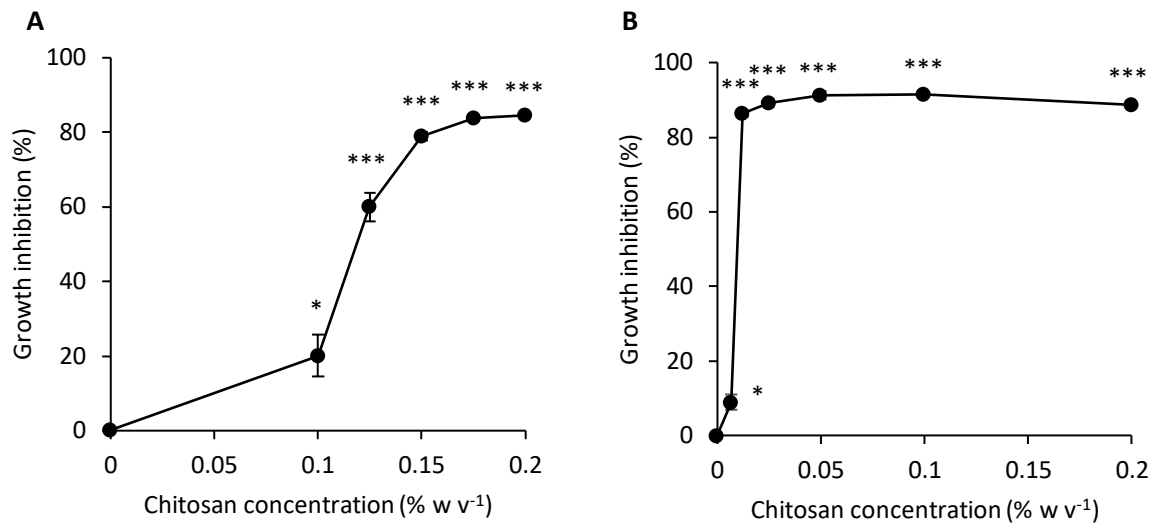

**Supplementary Figure S1:** *In vitro* growth inhibition of *F. graminearum* GZ3639 by chitosan.

Supplement: Supplementary file 1 — Additional file 1: Figure S1. In vitro growth inhibition of F. graminearum GZ3639 by chitosan. (A) Potato Dextrose Agar (PDA) was prepared with chitosan to final concentrations of 0.0, 0.1, 0.125, 0.15, 0.175 or 0.2% (w v-1) and was inoculated with a plug of F. graminearum GZ3639. The percentage of growth inhibition was calculated after 6 days of incubation based on the mycelial growth diameter measured. (B) Potato Dextrose Broth (PDB) was prepared with chitosan to final concentrations of 0.0, 0.00675, 0.0125, 0.025, 0.05, 0.1 or 0.2% (w v-1) and was inoculated with spores of F. graminearum. The percentage of growth inhibition was calculated after 4 days of incubation based on optical density measured. Error bars represent the standard error of the means. Asterisks above the data sets indicate that the data are statistically significantly different from the mock water treatment, according to a one-way ANOVA test (* = P ≤ 0.05, ** = P ≤ 0.01, *** = P ≤ 0.001). [file 12870_2022_3451_MOESM1_ESM.pdf]
