# Supplementary material for: The impact of chitosan on the early metabolomic response of wheat to infection by Fusarium graminearum
Source: BMC Plant Biol. 2022 Feb 19;22:73. doi: 10.1186/s12870-022-03451-w (PMC8857839; doi:10.1186/s12870-022-03451-w)
Supplement: Supplementary file 2 — Additional file 2: Figure S2. Box plot distribution of the effect of spraying wheat heads with chitosan on the percentage of spikelets infected with F. graminearum. The heads of wheat cv. Remus were sprayed at mid-anthesis with water (control) or 0.2% chitosan (w v-1) and were spray-inoculated 24 hours before (A) or after (B) with 0.02% Tween 20 (mock) or 105 spores ml-1 F. graminearum GZ3639. Asterisks above the data sets indicate that the data are statistically significantly different from the mock water treatment, according to Kruskal-Wallis tests (*** = P ≤ 0.001). [file 12870_2022_3451_MOESM2_ESM.pdf]

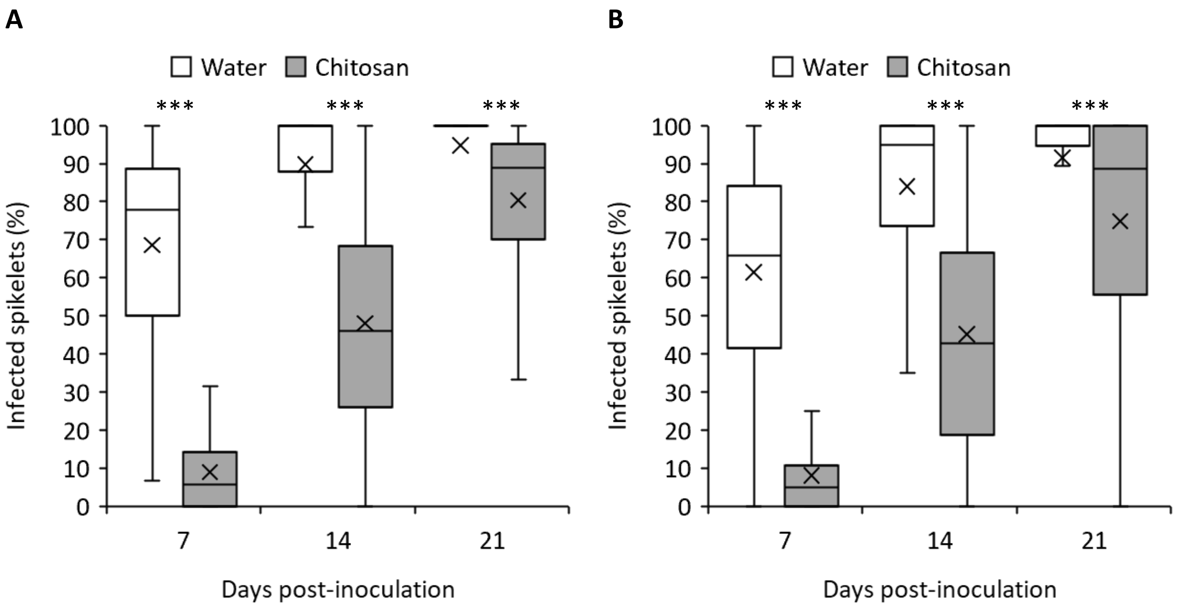

Supplementary Figure S2: Box plot distribution of the effect of spraying wheat heads with chitosan on the percentage of spikelets infected with *F. graminearum*.
