# Supplementary material for: The impact of chitosan on the early metabolomic response of wheat to infection by Fusarium graminearum
Source: BMC Plant Biol. 2022 Feb 19;22:73. doi: 10.1186/s12870-022-03451-w (PMC8857839; doi:10.1186/s12870-022-03451-w)
Supplement: Supplementary file 3 — Additional file 3: Figure S3. Representation of the Volcano plots delineating the features of interest. The features of interest were selected based on the fold change (FC, cut-off value 1.5) and the P value of the t-tests (P ≤ 0.05) performed to compare two sample categories. The samples corresponding to the 6- and 24-hour time points were pooled together for the statistical analysis. Three comparisons were performed: (A) chitosan (samples W_T compared with C_T, features regulated by chitosan), (B) F. graminearum (samples W_T compared with W_F, features regulated by F. graminearum) and (C) chitosan + F. graminearum (samples W_T compared with C_F, features regulated by the combination of chitosan and F. graminearum). The graphs represent the features that were significantly up- (red) or down-regulated (blue) or not selected for subsequent analysis (grey). [file 12870_2022_3451_MOESM3_ESM.pdf]

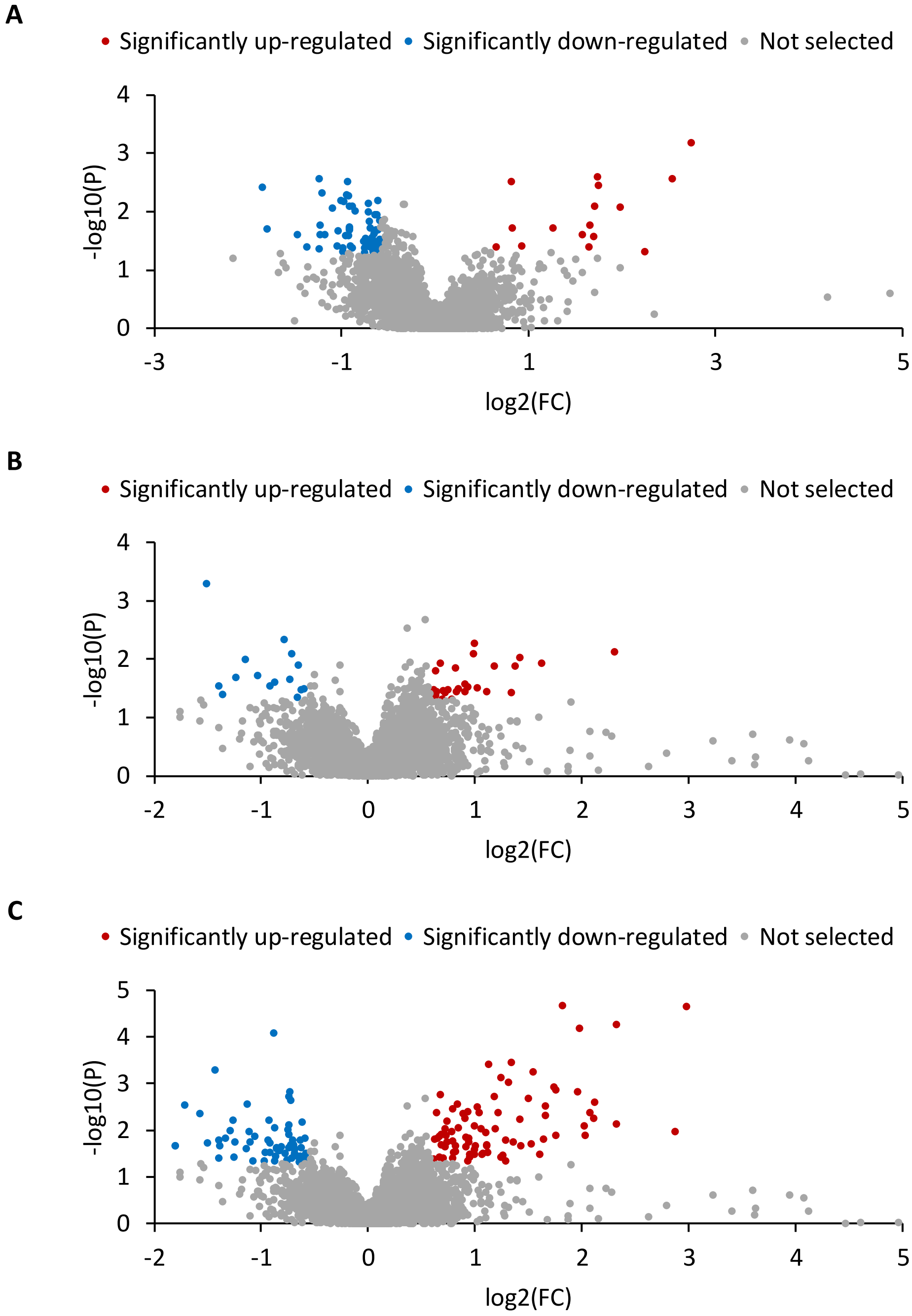

Supplementary Figure S3: Representation of the Volcano plots delineating the features of interest.
