# Supplementary material for: The impact of chitosan on the early metabolomic response of wheat to infection by Fusarium graminearum
Source: BMC Plant Biol. 2022 Feb 19;22:73. doi: 10.1186/s12870-022-03451-w (PMC8857839; doi:10.1186/s12870-022-03451-w)
Supplement: Supplementary file 4 — Additional file 4: Table S1. Total number of heads analysed (disease and yield component analysis) across the three replicate Fusarium head blight trials. [file 12870_2022_3451_MOESM4_ESM.docx]

**Table S1**: Total number of heads analysed (disease and yield component analysis) across the three replicate Fusarium head blight trials.

| **Experiment and treatment** | **Trait** | |
| --- | --- | --- |
|  | **Disease assessment** | **Yield component analysis** |
| **Pre-*Fusarium* treatment with chitosan** | | |
| water-*Fusarium* | 101 | 62 |
| chitosan-*Fusarium* | 109 | 62 |
| Water-mock | 100 | 60 |
| Chitosan-mock | 100 | 60 |
| **Post-*Fusarium* treatment with chitosan** | | |
| *Fusarium-*water | 106 | 61 |
| *Fusarium-*chitosan | 102 | 60 |
| Mock-water | 100 | 60 |
| Mock-chitosan | 100 | 60 |

Note, at least 20 heads per each of five replica trials were used for disease assessment and per each of three replica trials for yield component analysis.
